# Hydrogel-Based Enzyme and Cofactor Co-Immobilization for Efficient Continuous Transamination in a Microbioreactor

**DOI:** 10.3389/fbioe.2021.752064

**Published:** 2021-11-04

**Authors:** Tadej Menegatti, Polona Žnidaršič-Plazl

**Affiliations:** ^1^ Faculty of Chemistry and Chemical Technology, University of Ljubljana, Ljubljana, Slovenia; ^2^ Chair of Microprocess Engineering and Technology—COMPETE, University of Ljubljana, Ljubljana, Slovenia

**Keywords:** cofactor co-immobilization, microreaction engineering, flow biocatalysis, transaminase, hydrogel

## Abstract

A microbioreactor was developed in which selected amine transaminase was immobilized together with the cofactor pyridoxal phosphate (PLP) to allow efficient continuous transamination. The enzyme and cofactor were retained in a porous copolymeric hydrogel matrix formed in a two-plate microreactor with an immobilization efficiency of over 97%. After 10 days of continuous operation, 92% of the initial productivity was retained and no leaching of PLP or enzyme from the hydrogel was observed. The microbioreactor with co-immobilized cofactor showed similar performance with and without the addition of exogenous PLP, suggesting that the addition of PLP is not required during the process. The space-time yield of the microbioreactor was 19.91 g L^−1^ h^−1^, while the highest achieved biocatalyst productivity was 5.4 mg mg_enzyme_^−1^ h^−1^. The immobilized enzyme also showed better stability over a wider pH and temperature range than the free enzyme. Considering the time and cost efficiency of the immobilization process and the possibility of capacity expansion, such a system is of great potential for industrial application.

## Introduction

Biocatalytic processes are becoming increasingly important in modern chemistry as they enable more environmentally friendly production of various value-added molecules. The use of biocatalysts reduces the waste generated in the process (E-factor), lowers energy consumption, allows operation at mild process conditions, often uses water as the reaction medium, and eliminates the need for metal-based catalysts, which are usually problematic (Sheldon and Woodley, 2018; Aguilar et al., 2019). The introduction of continuous operation in biocatalytic processes, i.e., flow biocatalysis, can also meet the requirements for sustainable process design and process intensification (Žnidaršič-Plazl, 2021). The main challenges in the industrial implementation of biocatalysts include improved biocatalyst stability, cofactor regeneration/retention, and more efficient reactor design (Bolivar and López-Gallego, 2020), all of which are considered in this work.

The use of microflow systems in biotechnology has increased dramatically in recent years, as microreactor technology offers several advantages over conventional methods, especially in establishing continuous, i.e., flow processes. A larger surface-to-volume ratio allows for improved mass and heat transport with better temporal and spatial control, while surface modifications and the use of nanostructured materials lead to a larger surface area for enzyme immobilization (Wohlgemuth et al., 2015; Žnidaršič-Plazl, 2017; Tamborini et al., 2018). Enzyme immobilization plays a key role in flow biocatalysis by allowing the catalyst to remain in the reactor while being continuously supplied with reagents. Immobilization often increases the stability of the enzyme and simplifies product work-up (Romero-Fernández and Paradisi, 2020; Sheldon et al., 2021). In biocatalytic processes where the cofactor regenerates itself during the reaction, its co-immobilization is of great importance as it eliminates the need for its exogenous addition and stabilizes the enzyme structure. Together with miniaturization, the long-term use of enzyme and cofactor contributes decisively to the intensification of biocatalytic processes (Žnidaršič-Plazl, 2021).

Amine transaminases are enzymes that have great potential for the industrial production of optically active amines that enable the synthesis of various classes of biologically active compounds such as agrochemicals and pharmaceuticals (Kelly et al., 2018). The mechanism of enzymatic transamination consists of two half-reactions in which the cofactor pyridoxal-5′-phosphate (PLP) is regenerated and the amine acceptor/donor is converted to the corresponding amine/ketone (Weber et al., 2017). PLP is responsible for binding the amine moiety in the transamination step and acts as an enzyme stabilizing additive at higher substrate concentrations (Park et al., 2013). In addition to the successful co-immobilization of enzyme and cofactor in flow-through systems, Andrade et al. (2014) reported the development of a 0.5-ml packed-bed reactor with polymeric methacrylate beads for entrapment of *Escherichia coli* cells containing amine transaminase and PLP. A very stable continuous synthesis of chiral amines in organic reaction media was demonstrated, preventing leaching of the PLP cofactor. Another self-sufficient transamination system was presented by the group of López-Gallego (Benítez-Mateos et al., 2018), in which the enzyme and PLP were co-immobilized on different porous supports or in cross-linked enzyme aggregates by adding polyethylenimine (PEI) to achieve electrostatic interactions of PLP with the supports and prevent PLP leaching. On the other hand, little success was achieved in the cell-free biocatalytic synthesis of L-pipecolic acid by co-immobilization of lysine-6-dehydrogenase and pyroline-5-carboxylate reductase with NAD^+^ in agarose resin using PEI, as leaching of the cofactor over time or rapid loss of catalytic efficiency of the system was observed at different applied ionic strengths (Padrosa et al., 2020).

One of the simplest and non-invasive techniques to immobilize biocatalysts is their entrapment in porous structures by cross-linking different (bio)polymers such as alginates (Onbas and Yesil-Celiktas, 2019), chitosan (Kim et al., 2017), or synthetic polymers such as polyvinyl alcohol (PVA) (Bajić et al., 2017). We have previously reported the use of a copolymer hydrogel of PVA and alginate to immobilize yeast cells. This copolymer showed improved chemical and mechanical stability compared to alginate hydrogel (Menegatti and Žnidaršič-Plazl, 2019; Menegatti et al., 2021). In this work, a microreactor with copolymer hydrogel was developed and tested for the continuous model biotransformation of (*S*)-α-methylbenzylamine [(*S*)- α-MBA] and pyruvate (PYR) to obtain acetophenone (ACP) and L-alanine (L-ALA). Immobilization efficiency, productivity, and stability of the microreactor were evaluated. To the best of our knowledge, this is the first report on hydrogel-based co-immobilization of a transaminase with a cofactor.

## Materials and Methods

### Materials

Sodium alginate (SA), phenylboronic acid (PBA), 2-[4-(2-hydroxyethyl)piperazin-1-yl]ethanesulfonic acid (Hepes) buffer *(S)*-α-methylbenzylamine (*(S)*-α-MBA), sodium pyruvate (PYR), acetophenone (ACP), L-alanine (L-ALA), and pyridoxal 5′-phosphate (PLP) were all from Sigma Aldrich (St. Louis, MO), CaCl_2_ was from Carlo Erba reagents (Milan, Italy), polyvinyl alcohol (PVA, MW = 13,000–23,000 Da) was purchased from Acros Organics (Morris Plains, NJ). The enzyme amine transaminase (*ATA-v1*) was provided by c-LEcta GmbH (Leipzig, Germany).

### Batch Enzyme Activity Measurement

If not stated otherwise, the enzyme activity assays were carried out using 0.1 mM of PLP and 0.81 mg ml^−1^ of free or immobilized enzyme, 40 mM equimolar solution of *(S)*-α-methylbenzylamine (*(S)*-α-MBA) and sodium pyruvate (PYR) in 20 mM Hepes buffer (pH 8.0) (Miložič et al., 2018). The assays were carried out in thermostated test tubes at 30°C stirred at 300 rpm. The specific activity was expressed in U mg_*ATA-v1*_^−1^, where U was defined as the amount of enzyme that catalyzes the synthesis of 1 µmol of ACP per min under described conditions. All assays were performed in at least 3 replicates.

For the evaluation of pH and thermal stability of the immobilized enzyme, 0.1 mM PLP and 0.81 mg ml^−1^ enzyme were co-immobilized in copolymer hydrogel beads prepared as described in *Enzyme and Cofactor Co-immobilization in a Copolymeric Hydrogel*. Both the immobilized and free enzyme (the latter together with 0.1 mM PLP) were incubated for 30 min in 20 mM Hepes buffer at pH values ranging from 5 to 10 and at 25°C or in the same buffer with pH 8 and at temperatures ranging from 40 to 70°C. After the incubation period, the enzyme was transferred to the reaction mixture and the residual enzyme activity was determined under the standard conditions described above and compared with the activity before the incubation, where the reagent solutions were kept at 25°C before the reaction. Besides, recovered activity was calculated from the observed activity of the immobilized enzyme compared to that of the total free enzyme activity (Sheldon and van Pelt, 2013).

To estimate the temperature effect on biotransformation with free enzyme, enzyme activity was measured under the above conditions but at varied temperatures. The temperature was controlled by a thermostated bath placed on a magnetic stirrer with temperature control (IKA®, Staufen, Germany) and it varied from 30 to 80°C.

### Enzyme and Cofactor Co-Immobilization in a Copolymeric Hydrogel

To prepare a copolymer solution, 8% (w/v) PVA and 2% (w/v) sodium alginate were dissolved in a demineralized water by mixing at 60°C. After cooling to room temperature, *ATA-v1* and PLP were dissolved in Hepes buffer at various concentrations and mixed with the copolymer solution at a ratio of 1:1 (v/v) to obtain various final enzyme (0.81 and 8.1 mg ml^−1^) and PLP final concentrations from 0.1 to 1 mM as indicated in the Results section. Separately, a crosslinking solution was prepared by dissolving 2% (w/v) of CaCl_2_ and 2% (w/v) phenylboronic acid (PBA) in demineralized water.

To test the pH and temperature stability of the immobilized enzyme, the copolymer solution mixed with *ATA-v1* and PLP in concentrations to yield final concentrations of 0.81 mg ml^−1^ and 0.1 mM, respectively, was added dropwise through the needle of 0.6 mm inner diameter into a crosslinking solution, which was continuously stirred to prevent agglomeration of the formed hydrogel beads. After 60 min of crosslinking, the hydrogel beads were washed with Hepes buffer and stored for further use.

**FIGURE 1:**
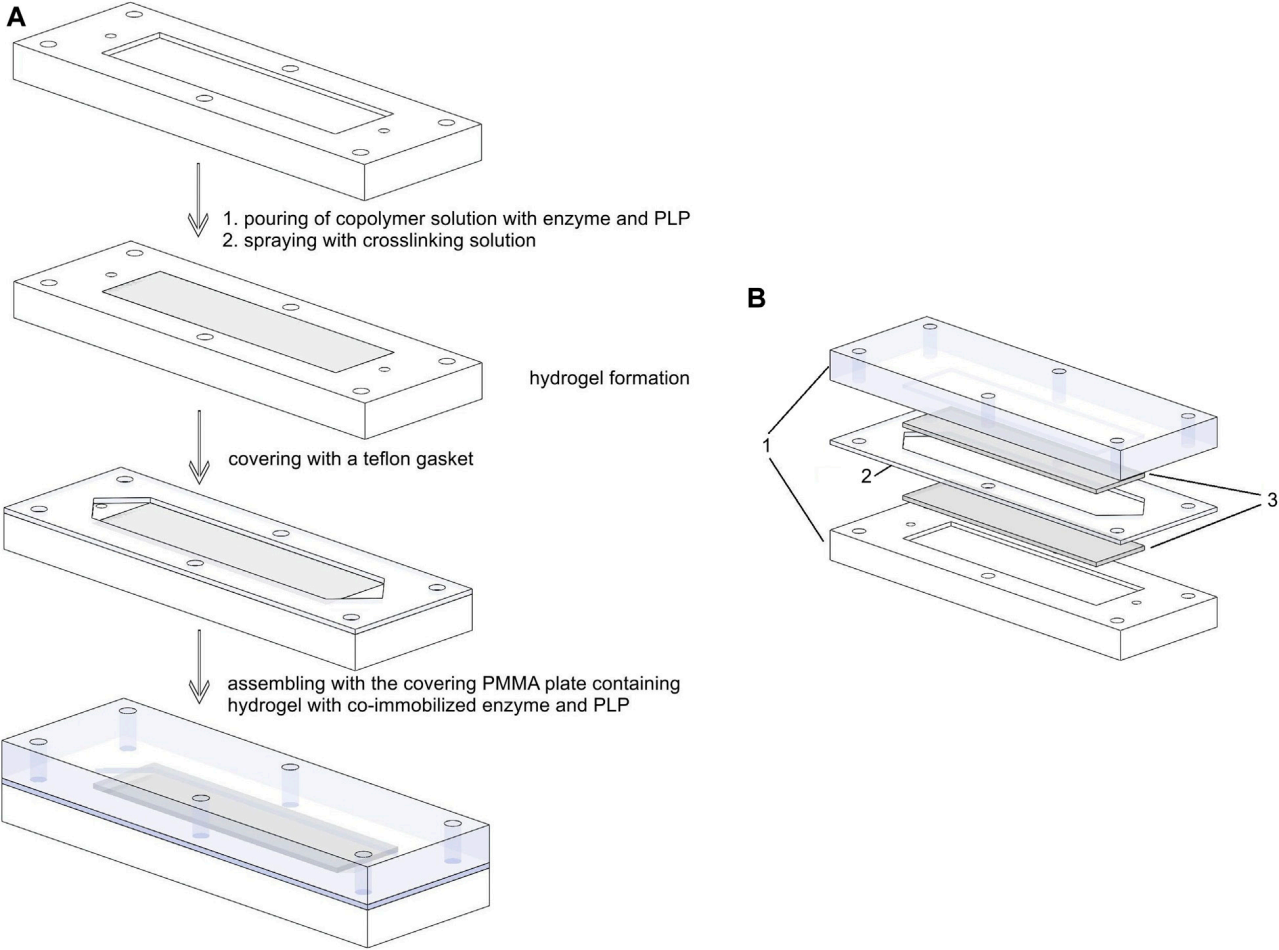
Schematic presentation of **(A)** immobilization procedure and assembly of a microbioreactor between two plates; **(B)** exploded view of a fully assembled microbioreactor: 1-PMMA plates; 2- PTFE gasket; 3-hydrogel layers with co-immobilized enzyme and PLP.

For immobilization in a microreactor, the mixed solution of enzyme, PLP, and copolymer was poured into the rectangular channel excavated into the PMMA plates. The crosslinking solution was sprayed onto the poured copolymer-buffer solution containing enzyme and PLP to initiate the crosslinking process (Figure 1A). After 60 min, the hydrogel layer formed was thoroughly washed with demineralized water before use.

### Microreactor Set-Up

The structure of the microreactor was described in our previous work (Menegatti and Žnidaršič-Plazl, 2019). Briefly, the microreactor consists of two poly (methyl methacrylate) (PMMA) plates serving as housings and separated by polytetrafluoroethylene (PTFE) spacers defining the geometry of the microchannels. In this setup, two PMMA plates had rectangular hollow chambers with a depth of 200 μm, a width of 25 mm, and a length of 50 mm, filled with a 200-µm thick hydrogel layer containing the biocatalyst and a cofactor. The distance between the two plates in the final assembly was determined by a 0.5-mm thick PTFE spacer, which was cut in the shape of a rectangular hydrogel with an additional triangular inlet and outlet part (Figure 1A), as previously described for the packed bed miniaturized reactors (Bajić et al., 2017; Strniša et al., 2018). The working volume of the microreactor with one inlet and one outlet shown in Figures 1A,B was measured to be 620 µL.

### Immobilization Efficiency in a Microreactor

Leakage of *ATA-v1* and PLP from the hydrogel embedded in a microreactor as described above was determined by measuring enzyme activity and PLP concentration in the samples collected at the outlet of the microbioreactor after Hepes buffer was pumped through the microbioreactor at the flow rate of 50 μL min^−1^. The initial enzyme concentration was 0.81 mg ml^−1^ or 8.1 mg ml^−1^, while the initial PLP concentration varied between 0.1 and 1 mM, as indicated in the Results section. Each sample corresponded to one reactor volume (620 µL). The activity assay was performed as described in *Batch Enzyme Activity Measurement* and the resulting solutions were analyzed by HPLC as described below, while PLP was quantified spectrophotometrically at 390 nm. Immobilization yield was calculated from the immobilized enzyme activity (the total activity in the outlet samples was subtracted from the starting activity) divided by the starting enzyme activity in the copolymeric solution. Recovered activity (%) was calculated from observed activity/free enzyme activity) (Sheldon and van Pelt, 2013).

### Continuous Transamination in a Microreactor

Unless otherwise indicated, an equimolar 40 mM solution of (*S*)-α-MBA and PYR in 20 mM Hepes buffer (pH 8.0) was pumped in a microbioreactor thermostated in a water bath at 50°C at flow rates ranging from 5 to 200 μL min^−1^ using a high-pressure pump from Harvard Apparatus (Holliston, MA) with a stainless-steel syringe (PHD 4400 Syringe Pump Series) (Bajić et al., 2017). The enzyme concentrations in the microreactor were 0.81 and 8.1 mg ml^−1^ (1.32 and 10.45 U ml^−1^, respectively), and the PLP concentrations varied from 0.1 to 1 mM, as indicated in the Results section. After reaching steady state, samples were taken from the microbioreactor outlet, quenched with 0.1 M NaOH, and further analyzed by HPLC as described below. Gross yields (%) were calculated based on ACP produced relative to (*S*)-α-MBA consumed. The space-time yield (STY) or volumetric productivity was calculated from ACP concentration in the sample taken from the microreactor outlet divided by the corresponding residence time. The values of STY were used to express the relative activity in case of temperature effect evaluation. Biocatalyst productivity was calculated from STY considering enzyme loading in the microbioreactor. Recovered immobilized specific activities [%] of the immobilized enzyme were calculated as the coefficient between the specific activity of the immobilized enzyme and the specific activity of the soluble one (Velasco-Lozano et al., 2020).

### Estimation of Temperature Effect on Biotransformation in a Microreactor

The effect of temperature on immobilized enzyme was evaluated in a microbioreactor with co-immobilized *ATA-v1* and PLP submerged into the thermostated water bath at the same temperatures as for the free enzyme. The reaction was performed as stated in *Continuous Transamination in a Microreactor* with 0.81 mg ml^−1^ enzyme and 0.1 mM PLP in the reactor, while the flow rate was set to 50 μL min^−1^ yielding mean residence time of 30 min. After reaching the steady state, outlet samples were collected and analyzed by HPLC as described below. Relative activities (%) were calculated from the results obtained at tested temperature as compared to the ones obtained at 50°C.

### Operational Stability Measurement

The operational stability of the microbioreactor system was evaluated by performing a continuous biotransformation for several days at 50°C. The reaction mixture with or without exogenously added PLP was pumped through the microbioreactor containing the co-immobilized *ATA-v1* and PLP at a flow rate of 2 μL min^−1^ for a period of 10 days. The experiment without exogenously added PLP was also performed at 30°C. Samples from the microbioreactor outlet were taken daily and analyzed by HPLC as described below. Operational stability (%) was expressed as relative volumetric productivity compared to the one at the start of the experiment.

### HPLC Analysis

The concentrations of (*S*)-α-MBA and ACP were determined by HPLC analysis. An HPLC with diode array detector (Shimadzu, Tokyo, Japan) and a Gemini-NX 3 µm C18 110 Å (150 × 4.60 mm) column (Phenomenex, Torrance, United States) was used for separation and detection of compounds with the isocratic method. The mobile phase consisted of 50% (v/v) acetonitrile and 50% (v/v) of 0.1 M aqueous NaOH solution at pH 11. The flow rate through the column thermostated at 30°C was 1 ml min^−1^. The retention times for (*S*)-α-MBA and ACP measured at 260 nm were 2.6 and 3.4 min, respectively.

## Results and Discussion

### Stability of Free and Immobilized Transaminase at Various pH Values and Temperatures

The pH stability of the free and immobilized transaminase was compared by measuring the enzyme activity after incubation at 25°C for 30 min in Hepes buffer at different pH values between 5 and 10. The enzyme was immobilized together with the PLP cofactor in copolymer hydrogel beads of approximately 1 mm in size, which were prepared by dropwise addition of a copolymer solution containing enzyme and cofactor into a crosslinking solution. The activity of free enzyme at standard conditions measured at 30°C in a batch process was 1.7 U/mg, while for the immobilized enzyme, the activity was 1.08 U/mg. Calculated recovered activity of immobilized transaminase was 63%, which is promising regarding literature reports. Up to 40% activity was recovered for *Pseudomonas putida* ω-transaminase immobilized in cross-linked enzyme aggregates (CLEAs) or by other immobilization techniques on different pre-existing matrices, except for entrapment in silica with 87% recovered activity (Velasco-Lozano et al., 2020).

**FIGURE 2:**
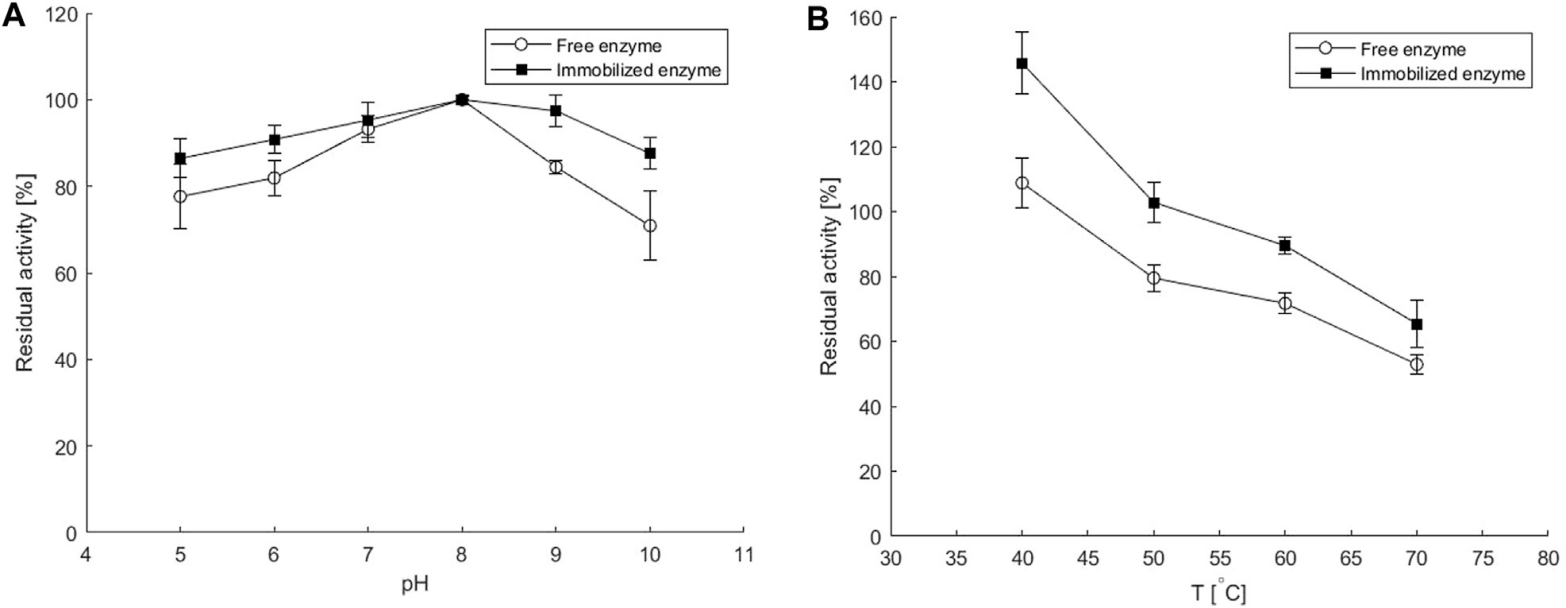
Residual activities of the free and the immobilized enzyme as calculated from the batch enzyme activity assays performed before and after a 30-min incubation at various **(A)** pH values and **(B)** temperatures at conditions specified in *Batch Enzyme Activity Measurement* and *Enzyme and Cofactor Co-immobilization in a Copolymeric Hydrogel*. Error bars indicate standard deviations of triplicates.

As can be seen in Figure 2A, the immobilized *ATA-v1* retained over 80% of the initial activity in the pH range of 5–10, while the free enzyme had much lower residual activity at pH values below or above pH 8. This is consistent with the results obtained for His-tagged ω-transaminase immobilized on magnetic mesoporous silica nanospheres functionalized with Ni^2+^-nitrilotriacetic acid, where immobilization significantly improved thermal and pH stability (Cao et al., 2017). The better stability of the immobilized enzyme in a wider pH range than that of the free transaminase could be due to electrostatic interactions of the carrier with the main fluid. Alginate and polyvinyl alcohol have negative charges on their surface, which leads to the attraction of H^+^ ions and results in a less alkaline microenvironment in the porous hydrogel than in the main solution (Cao et al., 2017).

The thermal stability of the immobilized *ATA-v1* also showed higher retained activities after 30-min incubation period at elevated temperatures up to 70°C, as shown in Figure 2B. At 70°C, the co-immobilized *ATA-v1* retained almost 70%, whereas the free form of the enzyme retained only about half of its initial activity at this temperature. Similar findings were reported for co-immobilization of *P. putida* ω-transaminase and PLP in CLEA with the addition of PEI, where the residual activity after 30 min at 70°C was 70%, but was much lower in the case without PLP co-immobilization (Velasco-Lozano et al., 2020). On the other hand, immobilization of the same enzyme by entrapment in silica having 87% recovered activity resulted in only 5% residual activity after 30 min incubation at 70°C (Velasco-Lozano et al., 2020).

The increased stability of the enzyme could be related to the presence of PLP during incubation, as the cofactor stabilizes the quaternary structure of the enzyme and maintains the essential interactions between the cofactor and the catalytic pocket. Moreover, the interactions between the alginate and PVA surface with the enzyme increase the resistance to higher temperatures in the microenvironment within the hydrogel matrix.

### Immobilization Efficiency

**FIGURE 3:**
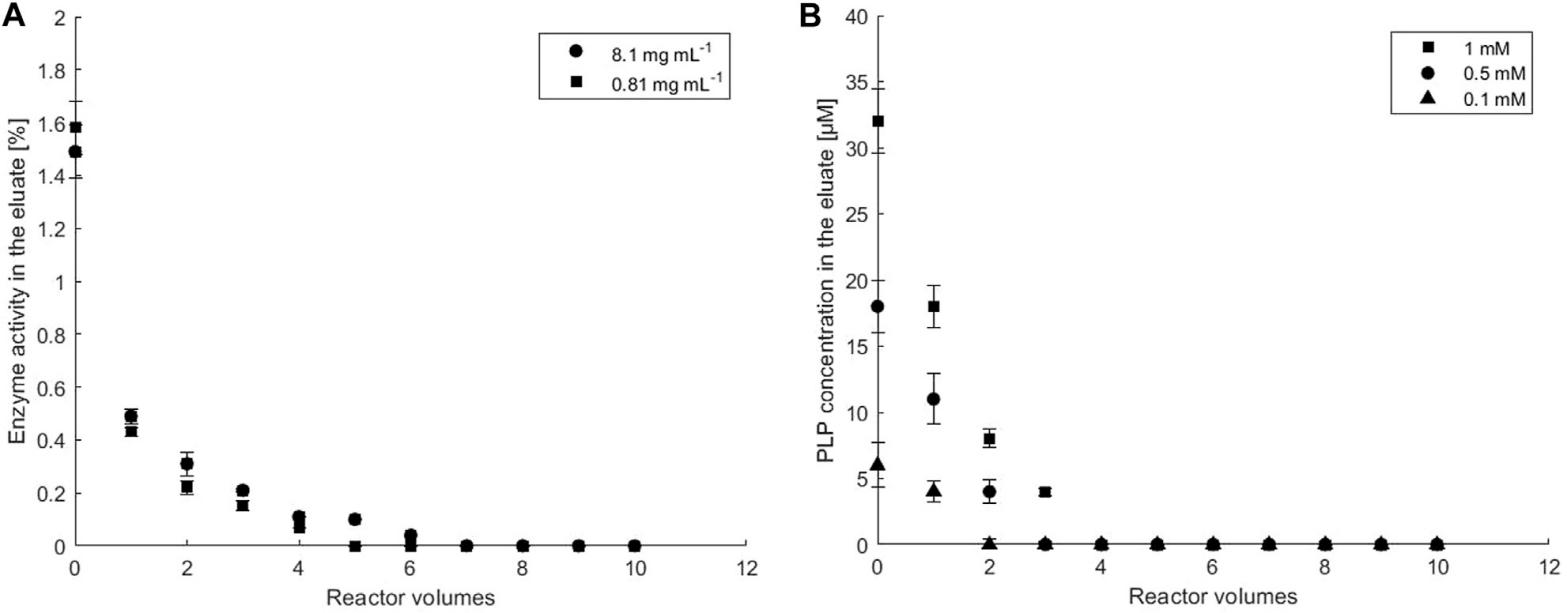
The stability of *ATA-v1* and PLP immobilization in a hydrogel embedded in a microbioreactor. **(A)** Leakage of *ATA-v1* from the microreactor at initial enzyme concentration stated in the legend and at 0.1 mM PLP, and **(B)** leakage of immobilized PLP cofactor from the microreactor at various initial concentrations of immobilized PLP stated in the legend, and at 0.81 mg ml^−1^ *ATA-v1*. The flow rate of the eluent (Hepes buffer, pH 8) was set to 50 μL min^−1^. Error bars indicate standard deviations of triplicates.

Immobilization efficiency for *ATA-v1* and PLP in the porous hydrogel matrix embedded in the microreactor assembly shown in Figure 1B was tested by pumping Hepes buffer through the microbioreactor and monitoring the leakage of both molecules at the microreactor outlet. After initial washout of the unconfined enzyme within the first 4 reactor volumes, *ATA-v1* activity in the outlet samples remained close to zero (Figure 3A), confirming that the enzyme was successfully retained in the porous matrix of the copolymer hydrogel. The immobilization yield taking into consideration leaking of the enzyme was above 97% for both tested enzyme concentrations. As for the cofactor co-immobilization, starting PLP concentrations of 0.1, 0.5, and 1 mM in the reactor were tested at the initial enzyme concentration of 0.81 mg ml^−1^. From Figure 3B after the initial washout of the excess PLP during continuous operation, no PLP leakage was detected in the effluent at all PLP concentrations tested. Total washout varied between 6.2% for 1 mM and 8.8% for 0.1 mM PLP. Since the smallest amount of unbound PLP was observed at a PLP concentration of 0.1 mM, this concentration was used in all further experiments.

For immobilization of enzymes based on hydrogels entrapment, its efficiency is mainly related to the hydrogel’s pore size and structure, which need to prevent the leakage of the biocatalyst. Recently, successful fixation of yeast cells in a copolymeric sodium alginate-PVA hydrogel was reported (Menegatti and Žnidaršič-Plazl, 2019), but immobilization of enzymes with molecular weight below 100 kDa having diameters up to 6 nm (Erickson, 2008) resulted in poor performance due to enzyme leakage (data not shown). These findings are consistent with the literature data on the average pore size of alginate hydrogels prepared from 2% (w/v) sodium alginate and 2% (w/v) CaCl_2_, which was defined to be 5 nm (Kopač et al., 2021), while 8% addition of PVA even reduced the average pore size below 4 nm (Menegatti et al., 2021). Since the tetrameric amine transaminase used in this study has a molecular weight of 194 kDa (Börner et al., 2017) corresponding to >8 nm protein diameter (Erickson, 2008), this explains successful retention of this enzyme together with a cofactor within the selected copolymer hydrogel.

Besides lowering the average pore size, the addition of PVA to alginate also improved chemical stability of the copolymer hydrogels compared to commonly used alginate hydrogels, making them more suitable for enzyme immobilization (Nunes et al., 2016; Menegatti and Žnidaršič-Plazl, 2019; Menegatti et al., 2021). Furthermore, PVA addition solves the problem encountered by calcium alginate hydrogels that are unstable in solutions with pH values higher than the *pKa* of their substituent groups, as such conditions disrupt the crosslinked 3D structure (Kopač et al., 2021), which renders them non-applicable for biotransformations such as aminations with amine transaminases, where the amine donors have high *pKa* values [e.g., *pKa* of (*S*)-α-MBA is 9.7].

### Biotransformation in a Microbioreactor

**FIGURE 4:**
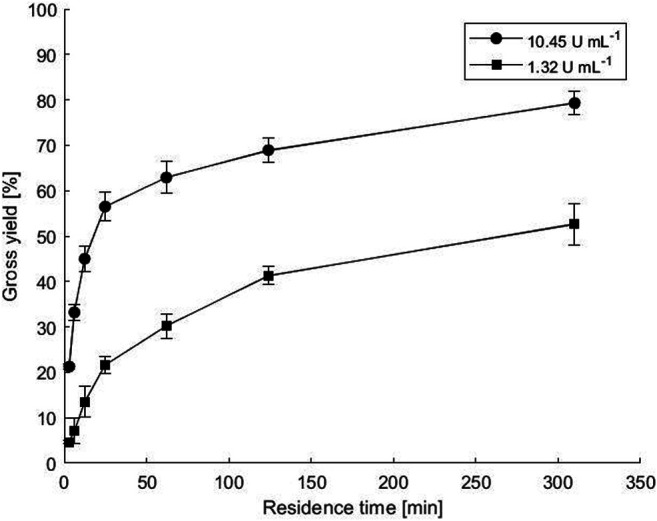
Biotransformation in a microbioreactor between two plates with co-immobilized *ATA-v1* and PLP in copolymer hydrogel. The points represent the gross yield of ACP as a function of residence time. Reactions were performed at various immobilized enzyme concentrations with 0.1 mM PLP cofactor at 50°C and with equimolar substrate concentrations of 40 mM. Error bars indicate standard deviations of triplicates.

Transamination in a microbioreactor was performed with two hydrogel layers between two plates, which has also been shown to be advantageous for fumaric acid transformation with yeast cells (Menegatti and Žnidaršič-Plazl, 2019). Two different enzyme loads were used, resulting in final enzyme activities of 1.32 and 10.45 U mL^−1^, while PLP was added at a final concentration of 0.1 mM. The calculated gross reaction yields in samples taken at the microbioreactor outlets, where the reaction mixture was pumped at various flow rates and thus residence times, are shown in Figure 4. As expected, higher enzyme load and longer residence times resulted in higher yields under the same conditions. The highest gross yield of 80% was obtained with a higher enzyme load and a residence time of 310 min, while a 10 times lower enzyme load at the same residence time resulted in a gross yield of 52%. Recovered activities calculated at the highest flow rate giving 3.1 min residence time were 44.8 and 26.4% for enzyme loads of 1.32 and 10.45 U mL^−1^, respectively.

The highest space-time yields (STY) calculated at the shortest residence time of 186 s were 4.13 and 19.91 g_ACP_ L^−1^ h^−1^ for enzyme loading of 1.32 and 10.45 U mL^−1^, respectively. This is much lower than reported for amine transaminase immobilized onto polymer-coated glass beads with controlled porosity (EziG™) via iron cation affinity binding. When tested in a packed bed reactor with a high enzyme loading of 112 mg ml^−1^, STY of 335 g_ACP_ L^−1^ h^−1^ was calculated at 54 s residence time (Böhmer et al., 2019). Also Bajić et al. (2017) reported a STY value of 277 g_ACP_ L^−1^ h^−1^ at 22 s residence time for the same reaction performed in a miniaturized packed bed reactor. On the other hand, surface immobilization in microreactors with amine transaminase genetically fused to a silica-binding Z_basic2_ protein (*N-SBM-ATA-wt*) in a silicon/glass microchannel yielded STY of only 14.42 g_ACP_ L^−1^ h^−1^, mainly due to the small enzyme loading of 1.57 U mL^−1^ that was possible on the reactor wall surface (Miložič et al., 2018).

When comparing biocatalyst (or specific) productivities calculated at the highest STY, 5.4 and 2.6 mg_ACP_ mg_ATA_^−1^ h^−1^ were obtained at enzyme loads of 1.32 and 10.45 U mL^−1^, respectively. This is comparable to 5.6 mg_ACP_ mg_ATA_^−1^ h^−1^ obtained with *N-SBM-ATA-wt* transaminase immobilized on the microchannel surface (Miložič et al., 2018), but again lower than in a packed bed reactor with transaminase immobilized on the surface of the beads, where a biocatalyst productivity of 244 mg_ACP_ mg_ATA_^−1^ h^−1^ was reported (Benítez-Mateos et al., 2018). This productivity was obtained at very high flow rates of 1.45 ml min^−1^ in an optimized packed bed reactor with *His*-tagged amine transaminase. In contrast, amine transaminase entrapped in lenticular *Lentikats* *®* PVA particles packed in miniaturized packed bed reactors resulted in lower specific productivities ranging from 0.17 to 16 mg_ACP_ mg_ATA_^−1^ h^−1^ (Bajić et al., 2017).

### Estimation of Temperature Effect on Biotransformation

The effect of temperature on transamination with immobilized enzyme was studied at flow conditions in a microbioreactor and compared to reaction with free enzyme defined in a batch process. The residence times in the microreactor and batch process times were identical.

**FIGURE 5:**
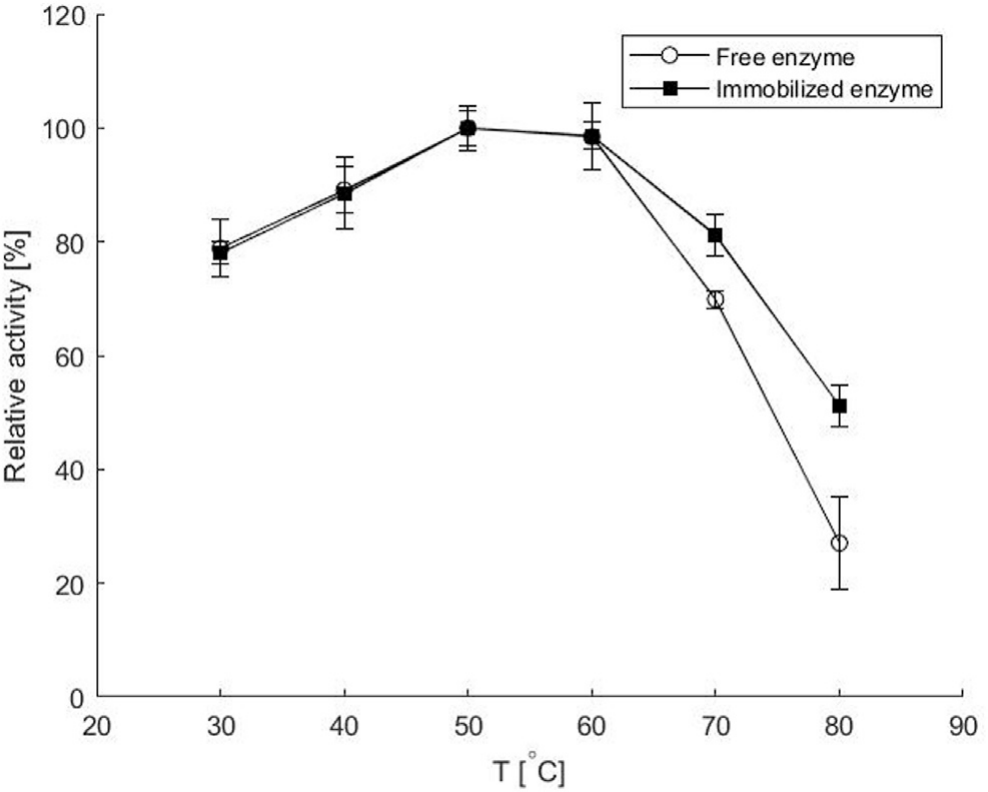
Relative activity of the free and the immobilized enzyme at various temperatures as compared to the reaction rate at 50°C. Experiments with immobilized enzyme were performed in a microreactor at 50 μL min^−1^ flow rate, while the reaction rate with free enzyme was determined using standard batch enzyme activity measurement. Error bars indicate standard deviations of triplicates.

From Figure 5 it is evident that increasing the reaction temperature up to 50°C had a positive effect on the reaction rate for both the free and immobilized enzyme and almost reached a plateau up to 60°C. This is comparable to the optimal temperatures reported for other amine transaminases from various organisms (Mallin et al., 2014). Further increase in temperature resulted in a decrease in reaction rate, which was much more pronounced when the free enzyme was used. At 80°C, the reaction with the immobilized enzyme was almost twice as fast as with the free enzyme, reaching only 26% of the maximum value for the latter. This is consistent with previous findings of improved thermal stability of the immobilized enzyme, as shown in Figure 2B, and with other literature reports. Thus, immobilization of bovine heart ω-transaminase in a sol-gel matrix even shifted the apparent temperature optimum and improved biotransformation rates compared to the reaction with free enzyme at temperatures up to 60°C. The good activity of the immobilized enzyme even at high temperatures could be due to the additional multi-point contact through hydrogen bonding as well as ionic interactions of the enzyme with the matrix (Koszelewski et al., 2010).

### Determination of Operational Stability

**FIGURE 6:**
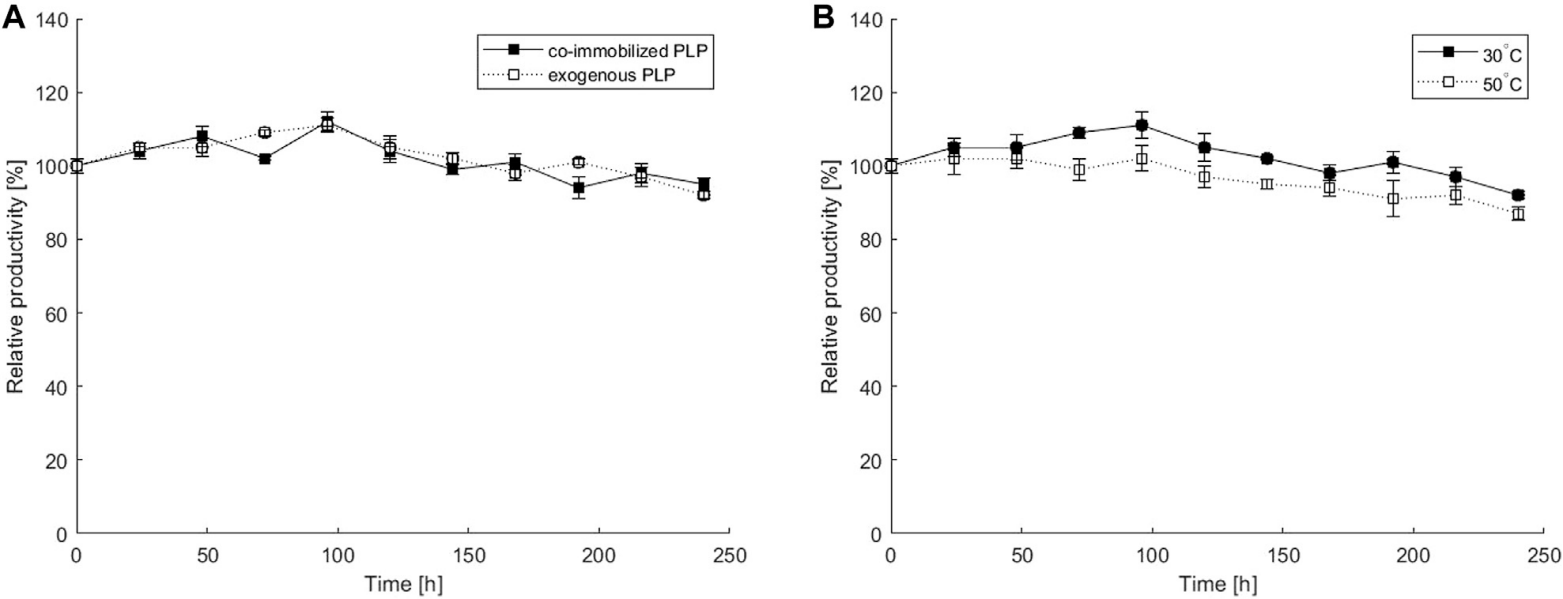
Operational stability of a microbioreactor over a 10-day period: **(A)** estimated at 30°C with 0.1 mM PLP co-immobilized in a hydrogel or exogenously added in a fluid stream; **(B)** estimated for 0.1 mM PLP co-immobilized in a hydrogel at 30°C or 50°C. All experiments were performed at 2 μL min^−1^ flow rate and with an equimolar substrate concentration of 40 mM and *ATA-v1* concentration of 0.81 mg ml^−1^. The initial STY at 30 and 50°C were 15.2 U g^−1^ and 19.4 U g^−1^, respectively. Error bars indicate standard deviations of triplicates.

To determine the operational stability of the co-immobilized system, continuous transamination was performed in a microbioreactor over a 10-day period. The process with co-immobilized *ATA-v1* and PLP, where only substrates were pumped through the reactor at a constant flow of 2 μL min^−1^, was compared with the system with immobilized *ATA-v1* and exogenously added 0.1 mM PLP in the reaction mixture, both run at 30°C. Figure 6A represents the relative productivity of the reactor over time for the system with immobilized PLP and the system with exogenously added PLP. The productivity fluctuates over a 10-day period without any significant loss. The microbioreactor with co-immobilized PLP retained 92% of its initial productivity after 10 days and showed similar performance to the reactor without immobilized but exogenously added PLP, confirming that co-immobilization of the cofactor was also successful. The microbioreactor with co-immobilized PLP was also tested for its operational stability at an optimal temperature of 50°C. As evident from Figure 6B, the relative productivity remained very stable during the 10-day continuous transamination and retained 87% productivity at the end of the experiment. The results obtained with co-immobilization in a copolymer hydrogel were much better than those reported for a silicon/glass microreactor with surface immobilized *N-SBM-ATA-wt* tested over a period of 4 days, with productivity dropping below 50% after only 16 h of continuous operation (Miložič et al., 2018). Similarly, (Abdul Halim et al., 2013) reported on an immobilized His-tagged transaminase packed in a microreactor, where the relative productivity also dropped to 60% after only 8 h of continuous transamination. On the other hand, the aforementioned lenticular PVA *Lentikats* ^*®*^ particles with immobilized amine transaminase in a miniaturized packed bed reactor showed great operational stability with about 80% retained productivity after 21 days of operation at room temperature (Bajić et al., 2017), suggesting that enzyme immobilization by entrapment also improves the operational stability of this enzyme.

The industrial potential of alginate hydrogel-based biocatalyst immobilization in a bio-lamina bioreactor for the conversion of methane into liquid fuel has been recently described (Jovanovic et al., 2018; Jovanovic et al., 2021). The identified features of such systems are relatively high biocatalyst loads with the ability to adjust the thickness and porosity of the film to allow efficient diffusion of substrates and product to the active sites.

## Conclusion

A successful co-immobilization of PLP cofactor with *ATA-v1* in porous copolymeric hydrogel that stabilizes the enzyme over a wide pH and temperature range is presented. This novel co-immobilization method does not require organic solvents or additional polycationic polymers for successful PLP retention in a flow-through microbioreactor. The developed microbioreactor between two plates enabled continuous operation without significant leaching of *ATA-v1* and PLP. The microbioreactor system also showed high operational stability over a period of 10 days, even at 50°C.

## Data Availability

The original contributions presented in the study are included in the article/Supplementary Material, further inquiries can be directed to the corresponding author.
